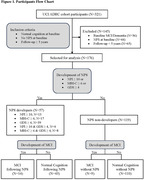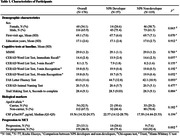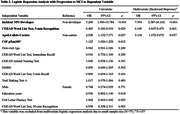# Association Between Incident Neuropsychiatric Symptoms and Cognitive Decline in Cognitively Unimpaired Older Adults

**DOI:** 10.1002/alz.091784

**Published:** 2025-01-03

**Authors:** Therese H Kim, Elizabeth Head, Craig EL Stark, Lorena Sordo, David L Sultzer

**Affiliations:** ^1^ The UC Irvine Institute for Memory Impairments and Neurological Disorders (UCI MIND), Irvine, CA USA; ^2^ School of Medicine, University of California, Irvine, Irvine, CA USA; ^3^ Department of Neurobiology and Behavior, University of California, Irvine, Irvine, CA USA

## Abstract

**Background:**

Neuropsychiatric symptoms (NPS) are highly prevalent in older adults. While the association between NPS and cognitive decline in older adults is widely acknowledged, there remains a lack of specificity regarding emerging NPS and cognitive outcomes in cognitively unimpaired older individuals. Our study assessed the incidence of NPS development, its link to cognitive decline, and other factors associated with development of MCI among cognitively unimpaired older adults without NPS at baseline.

**Method:**

Utilizing data from the UCI ADRC cohort, we included cognitively unimpaired participants without neuropsychiatric symptoms at baseline, followed longitudinally for at least 5 years. Comprehensive clinical assessments and expert diagnostic classifications occurred annually. NPS were evaluated using the Neuropsychiatric Inventory (NPI), the Mild Behavioral Impairment Checklist (MBI‐C), and the Geriatric Depression Scale (GDS). Demographic characteristics, cognitive test scores, ApoE genotype, CSF pTau181 level, and progression to MCI were compared between NPS developers and non‐developers. Logistic regression analysis, with progression to MCI as a dependent variable, was employed to explore factors influencing the likelihood of cognitive decline.

**Result:**

Participants were followed for an average of 8.4 years. The rate of NPS development was 32.4%, and 13.1% of participants progressed to MCI. There was no significant difference in demographic characteristics, baseline cognitive test scores, or biological markers between NPS developers and non‐developers. NPS developers exhibited a significantly higher rate of progression to MCI compared to non‐developers (24.6% vs. 7.6%, p = 0.002). In logistic regression analysis to predict progression to MCI, NPS development emerged as the most significant factor (OR = 5.209, p<0.001), followed by high CSF pTau181 level (OR = 1.122, p = 0.012), ApoE4 carrier status (OR = 2.928, p = 0.027), and low 5‐minute recognition score on the CERAD Word List Test (OR = 0.405, p = 0.029).

**Conclusion:**

These findings underscore the importance of assessing cognitive change in cognitively unimpaired older adults with recent‐onset NPS. Additionally, they indicate the potential benefits of combining NPS assessments with biological markers and cognitive test scores to predict those at risk of cognitive decline. These results further support the role of NPS in the pathophysiology of cognitive decline.